# OLTA: Optimizing bait seLection for TArgeted sequencing

**DOI:** 10.1093/bioinformatics/btaf146

**Published:** 2025-04-02

**Authors:** Mete Orhun Minbay, Richard Sun, Vijay Ramachandran, Ahmet Ay, Tamer Kahveci

**Affiliations:** Department of Computer Science, Colgate University, Hamilton, NY 13346, United States; Computer and Information Science and Engineering Department, University of Florida, Gainesville, FL 32611, United States; Department of Computer Science, Colgate University, Hamilton, NY 13346, United States; Departments of Biology and Mathematics, Colgate University, Hamilton, NY 13346, United States; Computer and Information Science and Engineering Department, University of Florida, Gainesville, FL 32611, United States

## Abstract

**Motivation:**

Targeted enrichment via capture probes, also known as baits, is a promising complementary procedure for next-generation sequencing methods. This technique uses short biotinylated oligonucleotide probes that hybridize with complementary genetic material in a sample. Following hybridization, the target fragments can be easily isolated and processed with minimal contamination from irrelevant material. Designing an efficient set of baits for a set of target sequences, however, is an NP-hard problem.

**Results:**

We develop a novel heuristic algorithm that leverages the similarities between the characteristics of the Minimum Bait Cover and the Closest String problems to reduce the number of baits to cover a given target sequence. Our results on real and synthetic datasets demonstrate that our algorithm, OLTA produces fewest baits for nearly all experimental settings and datasets. On average, it produces 6% and 11% fewer baits than the next best state-of-the-art methods for two major real datasets, AIV and MEGARES. Also, its bait set has the highest utilization and the minimum redundancy.

**Availability and implementation:**

Our algorithm is available at github.com/FuelTheBurn/OLTA-Optimizing-bait-seLection-for-TArgeted-sequencing. Test data and other software are archived at doi.org/10.5281/zenodo.15086636.

## 1 Introduction

Next-generation sequencing (NGS) technologies have greatly expanded our understanding of genomics by providing the capability for fast and cost-effective sequencing of biological samples. Despite these advancements, a significant portion of research continues to focus on specific sequences rather than the entire genetic composition of a sample. In such targeted studies, an overwhelming majority of NGS reads can originate from irrelevant sources. Research in areas such as antimicrobial resistance (AMR) (Noyes *et al.* 2016) and pathogen detection (Yang *et al.* 2011) have shown that up to 99% of raw NGS reads can come from nontargeted regions or entirely different organisms within a sample. Identification and removal of such irrelevant reads often require substantial efforts and computational resources (Hasan *et al.* 2016). Furthermore, the presence of such noise detrimentally impacts the sensitivity required for detecting low-abundance material (Hasan *et al.* 2016, Noyes *et al.* 2016, Lee *et al.* 2017).

For such applications, targeted enrichment with capture probes is a promising complementary procedure for conventional sequencing methods (Gnirke *et al.* 2009). Targeted enrichment uses short biotinylated oligonucleotide probes (hereinafter “baits”) to selectively isolate specific genomic regions of interest. Prior to sequencing, the baits are introduced to the sample and are given time to hybridize with complementary genetic material. Following hybridization, the hybridized fragments are isolated using magnets, allowing for easy removal of unhybridized material. The baits are then detached from the fragments, and the captured material is sequenced with minimal background interference. This procedure has found usage in numerous fields including phylogenomics (Andermann *et al.* 2019), pathogen detection (Gaudin and Desnues 2018, Pei *et al.* 2023), paleomicrobiology (Bos *et al.* 2019, Spyrou *et al.* 2019), and oncology (Singh 2022), and is predicted to expand to a broader range of applications in the near future (Quek and Ng 2024).

The success of targeted enrichment relies heavily on the bait set that is used in the procedure. An effective bait set must ensure comprehensive coverage of the targeted regions while minimizing the number of unique baits, as each unique bait adds to the manufacturing costs. Although complete coverage can be naïvely achieved by exhaustively listing every bait-length k-mer of the targeted regions as baits, this approach quickly becomes too costly with increasing target size. Additionally, baits can tolerate a number of mismatching base pairs when hybridizing with genetic material, allowing a single type of bait to hybridize with multiple similar yet nonidentical regions. Consequently, designing an optimal set of baits is a complex task, formulated as the NP-hard computational problem known as the Minimum Bait Cover problem (Alanko *et al.* 2022).

Most existing tools for designing baits limit their search space to the exact substrings of the input sequences. While this approach allows rapid bait design, it fails to exploit the fact that baits do not need to match the target sequence exactly to achieve effective hybridization. This unnecessary constraint leads to an excessive number of baits, substantially increasing sequencing costs. Indeed, recent results have shown that increasing bait length and reducing the hybridization temperatures allows for increased tolerance to mismatches (Ohrmalm *et al.* 2010, Miranda and Weber 2021). Recent literature reports up to 40 mismatch tolerance for baits of length 120 (Noyes *et al.* 2017) An alternative approach is to identify groups of similar regions within the input sequences and construct baits for these groups by solving the long-standing NP-hard Closest String problem (Kevin Lanctot *et al.* 2003). However, the only tool in our knowledge that uses this strategy (Chafin *et al.* 2018) requires multiple sequence alignments of the input sequences, making it unsuitable for applications where such resources are unavailable. Consequently, the prospect of using Closest String methods to address Minimum Bait Cover remains largely unexplored.

**Our contributions.** We develop an algorithm named *OLTA* (Optimizing bait seLection for TArgeted sequencing) that leverages the related Closest String problem. OLTA initially reduces the search space for potential bait matches by constructing a set of segment groups, ensuring that a bait set only needs to cover one segment from each group in order to cover the input sequences. The algorithm then uses a greedy strategy to identify a minimal set of baits that can cover a segment from each group. We compare OLTA to several existing methods, as reviewed in the next section, using both real and synthetic datasets. Our results on AIV and MEGARES datasets demonstrate that, on average across all mismatch allowances OLTA yields 6% and 11% reduction in the number of produced baits compared to closest existing methods while maintaining the third-shortest running time. OLTA is especially more effective on large mismatch allowances, which are harder-to-solve inputs. It produces 55% and 46% fewer baits than the second-best compared algorithm (CATCH and Syotti, respectively) on AIV and MEGARES datasets, respectively.

## 2 Background

Designing baits for targeted enrichment is a long-standing challenge that predates the formalization of the Minimum Bait Cover problem. In this section, we summarize the methods from the literature, acknowledging that some were developed to account for additional practical constraints (e.g. GC bias) that are not considered in the problem we address. We limit our experiments to the three most recently proposed methods which we describe below.

ProbeTools incrementally curates a set of baits using k-mer clustering (Kuchinski *et al.* 2022). In each iteration, ProbeTools uses a sliding window to enumerate bait-length substrings of the input sequences and clusters them based on sequence identity. It then adds the centroids of a user-specified number of largest clusters as baits to the solution set and identifies the regions of the input sequences that remain poorly covered by the current solution set. These poorly covered regions serve as the input sequences for the next iteration. This iterative process continues until the provided sequences are satisfactorily covered or a user-defined limit on the number of baits is reached.

The CATCH algorithm first generates a set of candidate baits by sliding a bait-length window across the input sequences (Metsky *et al.* 2019). For each candidate bait, it determines the coverage profile using a seed-and-extend heuristic while adhering to the hybridization constraints specified by the user for each input sequence. The algorithm then finds the smallest set of baits that can cover the sequences by reducing the problem to an instance of Minimum Set Cover. This is achieved by iteratively adding the candidate bait that covers the greatest number of uncovered positions to the solution set and updating the coverage profiles of the remaining candidates until the user-specified percentage of the sequences is covered.

The Syotti algorithm begins by constructing a generalized suffix array of the input target sequences (Alanko *et al.* 2022). It then initializes a bit vector with a length corresponding to the concatenation length of all input sequences. Each bit in this vector indicates whether a bait in the solution set covers that specific position in the sequences. The algorithm then carries out a single pass through this bit vector: whenever it encounters an uncovered position, it adds the bait-length substring starting at the corresponding position of the input sequence to the solution set. To identify the additional positions covered by the new bait, the algorithm uses a seed-and-extend heuristic using the generalized suffix array.

The following are additional tools developed for designing baits, but we do not include them in our comparisons for the reasons we now specify. MrBait (Chafin *et al.* 2018) and BaitsTools (Campana 2018) construct a bait set by using a tiling method, but are less recent than the algorithms we compare. MetCap (Kushwaha *et al.* 2015) uses ProDesign (Feng and Tillier 2007) to identify common substrings as candidate baits, but does not ensure complete coverage of input sequences. BaitMaker (Kamaraj *et al.* 2019) and AnthOligo (Jayaraman *et al.* 2020) design probes to be evenly spaced out across the target region, which is incompatible with our problem definition. BaitFisher (Mayer *et al.* 2016), HUBDesign (Dickson *et al.* 2021), supeRbaits (Jiménez-Mena *et al.* 2022), and PHYLUCE (Faircloth 2016) require additional resources alongside the input sequences and/or do not allow customization of the hybridization constraints, preventing them from being used for more generalized instances of Minimum Bait Cover.

## 3 Problem and algorithm specification

In the subsequent sections, we first introduce the necessary notation for this study and formally define the Minimum Bait Cover problem. Following this, we provide a detailed discussion of our algorithm.

### 3.1 Preliminaries and problem definition

For a given alphabet Σ, we define a string S as a finite sequence of characters over Σ. We denote the length of a string S as |*S*|. Using 0-based indexing, we denote the *i*th character of *S* as *S*[*i*], and the substring of S that spans from the ith character (inclusive) to the jth character (exclusive) as S[i…j]. Finally, we define the Hamming distance between two equal-length strings S and B, shown as d(S,B), as the number of indices at which they have different characters, formally d(S,B):=|{i:0≤i<|S| and S[i]≠B[i]}|.

We now provide the required definitions for Minimum Bait Cover (Alanko *et al.* 2022). Given two strings S and B, a positive integer θ, and a position i<|S|, we say that B  θ-covers a position i in S if there is a position j in S such that i−|B|<j≤i and d(S[j…j+|B|],B)≤θ. Using this definition, we say that a set of strings B  θ-covers a string S if every position i in S is θ-covered by some string in B. We formally define the Minimum Bait Cover problem as follows: Given a set of input strings S and integers L>0 and θ≥0, what is the smallest set B of L-length strings that θ-cover every position in S?

### 3.2 OLTA algorithm

First, we define several terms relevant to our algorithm. We define a bucket T as a set of L-length strings. We say that a string B  θ-matches a bucket T if there is at least one string T∈T that is θ-covered by B (this is equivalent to d(B,T)≤θ since |B|=|T|=L). Similarly, we say that a set of strings B  θ-matches a set of buckets $T^{*}$ if every bucket in $T^{*}$ is θ-matched by some string in B. Let $m(B,T^{*},\theta )$ denote the subset of buckets in $T^{*}$ that are θ-matched by B. It follows that if B  θ-matches $T^{*}$, then $m(B,T^{*},\theta )=T^{*}$.

In addition to the problem specification, our algorithm takes three additional inputs: (i) a positive integer $\omega <\left\lfloor \frac{L}{2}\right\rfloor$ representing the *bucket size* , (ii) a positive integer ϕ≤2θ representing the *lenient tolerance*, and (iii) a sequence of positive integers $K=K_{1},K_{2},\ldots K_{\left|K\right|}$ representing the *search breadths for bait generation*. We describe the purpose of each of these parameters in detail later in this section.

Algorithm 1 Main algorithm (OLTA)

**Input**: Set of input sequences S, bait length L, mismatch tolerance θ, bucket size ω, lenient tolerance ϕ, search breadths K

**Output**: Set of baits B that θ-cover all S in S

1: $T^{*}\leftarrow$ Initialize buckets (Algorithm 2)

2: **return**  B← Bucket covering (Algorithm 3)

Algorithm 2 Bucket initialization

**Input**: Sequences S, bait length L, bucket size ω<L

**Output**: Set of buckets $T^{*}$ such that θ-matching every $T\in T^{*}$ would θ-cover every S∈S

1: $T^{*}\leftarrow \emptyset$

2: **for all**  S∈S  **do**

3:   $T^{S}\leftarrow \emptyset$, $u_{S}\leftarrow \left\lceil \frac{\left|S\right|}{L-(\omega -1)}\right\rceil$

4:   **for**  j=2  **to**  $u_{S}-1$  **do**

5:    $\lambda_{j}^{S}=(j-1)(L-(\omega -1))$

6:    $T_{j}^{S}\leftarrow \{S[\lambda_{j}^{S}+i\ldots \lambda_{j}^{S}+i+L]:0\leq i<\omega \}$

7:    $T^{S}\leftarrow T^{S}\cup \left\{T_{j}^{S}\right\}$

8:   **end for**

9:   $T^{*}\leftarrow T^{*}\cup T^{S}\cup \left\{\left\{S[0\ldots L]\},\{S[|S|-L\ldots |S|]\right\}\right\}$

10: **end for**

11: **return**  $T^{*}$

Our algorithm runs in two main stages (see Algorithm 1). Below, we first provide an intuitive summary of how OLTA works. We then present the formal description.

1. The first stage takes the input sequences which need to be covered, and produces a set of buckets $T^{*}$. Each bucket corresponds to a set of bait length subsequences from the input data. The main goal for creating these buckets is that, they reduce the search space significantly as the number of buckets is typically much smaller than the number of nucleotides in the input data. OLTA creates these buckets in a way that ensures a solution set that θ-matches every bucket in $T^{*}$ would also θ-cover every sequence S in S. Furthermore, each bucket provides a signature of a potential bait which is guaranteed to cover all subsequences in that bucket.
2. The second stage of OLTA incrementally picks baits by utilizing these buckets until all buckets are covered. While there exists a bucket in $T^{*}$ that is not θ-matched by the current solution set, OLTA randomly selects such a bucket and use it to generate candidate baits. OLTA adds the most promising candidate to the solution set, specifically, the bait that θ-matches the maximum number of buckets. This stage makes two important observations, which dramatically reduces the number of baits in the final solution as compared to state-of-the-art algorithms. First, a bait which covers all the subsequences in a given bucket does not have to be one of the subsequences in the bucket, nor a subsequence from the input data. It instead creates a bait whose distance to the farthest subsequence in that bait is minimal. This is analogous to selecting the centroid of a set of points in a high dimensional Euclidean space. Second, it is possible that a bait, or a collection of baits which are already selected from a collection of buckets so far may coincidentally cover a bucket which has not been considered yet, thus further eliminating redundant baits.

In the first stage (Algorithm 2), we tile every input sequence S∈S with L-length substrings that overlap with each other in ω−1 positions. This results in $u_{S}=\left\lceil \frac{\left|S\right|}{L-(\omega -1)}\right\rceil$ substrings for each S (line 5). We denote the jth substring of S as $s_{j}^{S}$ and its starting position in S as $\lambda_{j}^{S}$, i.e. $s_{j}^{S}=S[\lambda_{j}^{S}\ldots \lambda_{j}^{S}+L]$. For $j<u_{S}$, let $\lambda_{j}^{S}=(j-1)(L-(\omega -1))$. For $j=u_{S}$, let $\lambda_{j}^{S}=|S|-L$; i.e. the end of $s_{u_{S}}^{S}$ is always aligned with the end of S (and is allowed to overlap with the previous substring $s_{u_{S}-1}^{S}$ in more than ω−1 positions). For each resulting substring $s_{j}^{S}$, we create a respective bucket $T_{j}^{S}$ that contains $s_{j}^{S}$. Additionally, for each position in which a substring $s_{j}^{S}$ overlaps with a previous substring $s_{j-1}^{S}$ (i.e. the first ω−1 positions of $s_{j}^{S}$), we insert the L-length substring that starts at the next position in S into the respective bucket $T_{j}^{S}$ (line 8). In summary, for $1<j<u_{S}$, $T_{j}^{S}=\{S[\lambda_{j}^{S}+i\ldots \lambda_{j}^{S}+i+L]:0\leq i<\omega \}$, and for $j\in \{1,u_{S}\}$, $T_{j}^{S}=\{s_{j}^{S}\}$: This is because $s_{1}^{S}$ does not overlap with a previous substring; and, $s_{u_{S}}^{S}$ is aligned with the end of S, so any substring $S[\lambda_{u_{S}}^{S}+i\ldots \lambda_{u_{S}}^{S}+i+L]=S[|S|-L+i\ldots |S|+i]$ would go out of bounds for i≥1. Our final set of buckets is $T^{*}=\{T_{j}^{S}:S\in S\;\text{and}\;1\leq j\leq u_{S}\}$. Creating the buckets in this way ensures that a solution set that would θ-match every $T\in T^{*}$ would also θ-cover every S∈S; we provide a proof of this claim in the Supplementary Material.

Algorithm 3 Bucket covering

**Input**: Buckets $T^{*}$, mismatch tolerance θ, lenient tolerance ϕ, search breadths K

**Output**: Solution set B that θ-matches $T^{*}$

1: $m(B,T^{*},\theta ):=\left\{T\in T^{*}:\exists T\in T\;s.t.\;d(B,T)\leq \theta \right\}$

2: B←∅

3: **while**  $T^{*}\neq \emptyset$  **do**

4:   $T\leftarrow \;\text{an}\;\text{arbitrary}\;\text{bucket}\;\text{in}\;T^{*}$

5:   **for all**  T in T  **do**  ▹ Compute |T| candidate baits

6:    $B_{T}\leftarrow$ Candidate bait from T (Algorithm 4)

7:   **end for**

8:   $B_{T}\leftarrow \mathrm{argma}x_{B_{T}:T\in T}\left|m(B_{T},T^{*},\theta )\right|$

9:   $B\leftarrow B\cup \left\{B_{T}\right\}$

10:   $T^{*}\leftarrow T^{*}\setminus m(B_{T},T^{*},\theta )$

11: **end while**

12: **return**  B

The second stage (Algorithm 3) incrementally computes a set of baits B that θ-matches the set $T^{*}$ produced in the first stage. We start with an empty solution set B:=∅. In each increment, we interpret the current $T^{*}$ as the set of buckets so far not θ-matched by B. As long as $T^{*}$ is nonempty, we pick an arbitrary bucket T from $T^{*}$ and proceed to θ-match T in the current increment, which requires θ-covering some string T∈T. To do this, we use Algorithm 4 to generate a candidate bait $B_{T}$ for each substring T∈T (creftypeplural 9, 8, and 7). Any such bait would make progress toward a solution that θ-matches $T^{*}$. Of all the candidates, we add to our solution set B the bait that θ-matches as many other buckets from $T^{*}$ as possible, i.e. the candidate $B\in {\left\{B_{T}\right\}}_{T\in T}$ such that $m(B,T^{*},\theta )$ is maximum (creftypeplural 11 and 10). We also remove those matching buckets from $T^{*}$ prior to the next increment (creftype 12). We then restart this process with another bucket until all buckets are matched.

Algorithm 4 Candidate bait production

**Input**: String T, buckets $T^{*}$, mismatch tolerance θ, lenient tolerance ϕ, search breadths K

**Output**: Candidate bait $B_{T}$

1: $\Phi_{T}\leftarrow \{T^{\prime }\in \cup T^{*}:d(T,T^{\prime })\leq \phi \}$, sorted by $d(T,T^{\prime })$

2: $B_{T}\leftarrow T$

3: $\Theta_{T}\leftarrow \{T^{\prime }:T^{\prime }\in \Phi_{s}\;\text{and}\;d(B_{T},T^{\prime })\leq \theta \}$

4: $g\leftarrow |\Theta_{T}|$

5: **for**i←1  **to**  ∞  **do**

6:   **for**j←1  **to**  $K_{i}$ (1 if i>|K|; skip if $\left|\Theta_{T}|=|\Phi_{T}\right|$) **do**

7:    $\Theta_{T}^{\prime }\leftarrow \Theta_{T}\cup \left\{{\left(\Phi_{T}\right)}_{g+j}\right\}$

8:    $B_{T}^{\prime }\leftarrow$WFC-CSP($\Theta_{T}^{\prime }$)

9:    **if**  $\max(\{d(B_{T}^{\prime },T^{\prime }):T^{\prime }\in \Theta_{T}^{\prime }\}\leq \theta )$  **then**

10:     $\Theta_{T}\leftarrow \Theta_{T}^{\prime },B_{T}\leftarrow B_{T}^{\prime }$, g←g+j

11:     **continue** next iteration at line 5

12:    **end if**

13:   **end for**

14:   **return**  $B_{T}$

15: **end for**

Algorithm 4 produces a candidate bait $B_{T}$ for a substring T∈T with a goal of θ-covering not only T but also strings in other unmatched buckets. First, we find the set $\Phi_{T}$ of substrings across all other unmatched buckets whose Hamming distance to T is at most ϕ, namely $\Phi_{T}=\{T^{\prime }\in \cup T^{*}\setminus \{T\}:d(T,T^{\prime })\leq \phi \}$. Since we limit ϕ≤2θ, $\Phi_{T}$ contains strings that could be θ-covered by a bait that also covers T. This is because, for any ϕ≤2θ and any substring $T^{\prime }\in \Phi_{T}$, we can construct a string B such that d(B,T)≤θ and $d(B,T^{\prime })\leq \theta$; conversely, for an arbitrary string $T^{\prime }$ (not necessarily coming from $\Phi_{T}$), if there is a B that satisfies d(B,T)≤θ and $d(B,T^{\prime })\leq \theta$, we can conclude $d(T,T^{\prime })\leq 2\theta$. Both these claims follow from the triangle-inequality property of Hamming distance, and we provide proofs in Supplementary Material. Ultimately, we want to construct a candidate bait $B_{T}$ such that its corresponding subset of θ-covered substrings $\Theta_{T}\subseteq \Phi_{T}$ includes substrings from as many different unmatched buckets as possible; we do so by considering different strings from $\Phi_{T}$ and constructing a bait that covers those strings and T. $\Phi_{T}$ thus forms a search space for this algorithm: With ϕ=2θ, $\Phi_{T}$ includes all strings that could be covered along with T; setting ϕ<2θ can reduce that number of strings and could result in a faster running time.

Rather than exhaustively trying all strings in $\Phi_{T}$, we follow an iterative greedy strategy to find a good bait $B_{T}$. We start by rewriting $\Phi_{T}$ as a sorted sequence $\Phi_{T}:=T_{1},T_{2},\ldots T_{\left|\Phi_{T}\right|}$ such that for $1\leq i<|\Phi_{T}|$, $d(T,T_{i})\leq d(T,T_{i+1})$ (creftype 3). We initialize the candidate $B_{T}$ as T itself and $\Theta_{T}$ as the set $\left\{T^{\prime }\in \Phi_{T}:d(B_{T},T^{\prime })\leq \theta )\right\}$ (creftypeplural 5 and 4). Let g be the greatest integer such that $T_{g}$ is included in $\Theta_{T}$. We try to expand $\Theta_{T}$ within |*K*| iterations. For each iteration i, we try, in order, each of the $K_{i}$ substrings that immediately follow the last element of $\Theta_{T}$ in the sequence $\Phi_{T}$ (i.e. $T_{g+1},T_{g+2},\ldots T_{g+K_{i}}$) as a possible addition to $\Theta_{T}$. For each substring $T_{g+j}$, we define $\Theta_{T}^{\prime }:=\Theta_{T}\cup \{T_{g+j}\}$ and use the Closest String heuristic WFC-CSP (Xu and Perkins 2022) to compute a string $B_{T}^{\prime }$ that is a Hamming-distance center for $\Theta_{T}^{\prime }$ (creftypeplural 10 and 9). If $B_{T}^{\prime }$ has a Hamming distance at most θ to every string in $\Theta_{T}^{\prime }$, we update $\Theta_{T}:=\Theta_{T}^{\prime }$, $B_{T}:=B_{T}^{\prime }$, and g:=g+j, and immediately start the next iteration $K_{i+1}$ (creftypeplural 13, 12, and 11). Otherwise, we retry the process with the next substring $T_{g+j+1}$ in the current iteration. If the current iteration’s final substring $T_{g+K_{i}}$ cannot be used to grow our subset, we halt our search and let the current bait $B_{T}$ be the candidate produced for T. If all |*K*| iterations result in a successful improvement, we assume that K has infinite trailing 1 s and carry out more iterations until we come across an unsuccessful iteration or $\left|\Theta_{T}|=|\Phi_{T}\right|$.

**Implementation details.** We implemented our OLTA algorithm in C++ and developed a custom analyzer in Python to evaluate the bait sets produced in our experiments. We provide the code of OLTA and our analyzer publicly (https://github.com/FuelTheBurn/OLTA-Optimizing-bait-seLection-for-TArgeted-sequencing). We provide details about our analyzer in Supplementary Material.

## 4 Results

In this section, we extensively evaluate the performance of OLTA. We begin by describing the experimental setup used for our analysis. Subsequently, we compare our algorithm with three existing approaches: ProbeTools, CATCH, and Syotti.

### 4.1 Experimental setup

#### 4.1.1 Datasets

We use two real datasets and one synthetic dataset.

*Real dataset 1.* AIV is a dataset of 11 967 neuraminidase genome segment reference sequences of avian-origin influenza A viruses, adding up to over 16 million base pairs. The dataset represents over 150 subtypes with sequences collected over a span of three decades. This dataset was curated by and ProbeTools and sourced from their GitHub repository. (Kuchinski *et al.* 2022).

*Real dataset 2.* MEGARES is a comprehensive database of antimicrobial resistant genes containing 8733 sequences that collectively total over 9 million base pairs (Bonin *et al.* 2023).

*Synthetic dataset.* We construct a database with synthetically generated sequences from the nucleotide alphabet Σ={A,C,G,T} stochastically to evaluate the performances of the algorithms in a controlled setting. We create sequences based on three parameters: (i) *SL*, the total length (number of nucleotides) of the sequence, (ii) *RN*, the number of unique repetitive short seed sequences embedded within the sequence, and (iii) *RC*, the fraction of the sequence composed of these seed sequences. We provide the pseudocode of the sequence generation algorithm in Supplementary Material. The generated sequences are publicly available alongside our analyzer (https://github.com/FuelTheBurn/OLTA-Optimizing-bait-seLection-for-TArgeted-sequencing).

#### 4.1.2 Evaluation criteria

We compare the algorithms based on the number of baits they produce and their running times. To estimate the produced baits’ effectiveness in practice, we also measure the following five criteria: (i) GC bias, (ii), homopolymer presence, (iii), repeat content, (iv) redundancy, which is a measure of the number of excess baits that cover each position in an input set, and (v) per-bait coverage, which is a probabilistic estimation of the number of positions that are expected to be covered by each produced bait. We provide the formal definitions of the latter two metrics in Supplementary Material.

#### 4.1.3 Experiment settings

We conduct five sets of experiments, two using the real datasets and three using the synthetic dataset, to investigate the performances of the algorithms with varying input parameters and properties. For all experiments, we set the bait length parameter to 120, for this bait length is recommended by the bait manufacturing company Agilent and is used in the literature (Noyes *et al.* 2017) including those studies we compare against (Alanko *et al.* 2022, Kuchinski *et al.* 2022). We provide details regarding the other parameters in the discussion of each experiment.

To maximize the candidate bait search space, we ran our algorithm with the additional parameter ϕ=2θ for each experiment. After experimenting with the K parameter, we determined that the values of 10,10,10,2 provided a good overall representation of our algorithm’s performance. Finally, we varied the ω parameter to demonstrate OLTA’s trade-off between minimizing the number of baits and reducing running time. We used ω=5 and 10 for our comparisons with the other algorithms. We provide the results of the other ω values in and.

We conducted all experiments on the Colgate University computing cluster (Colgate 2025). Each job was submitted to a single Linux worker node and was allocated 8 processors. We provide the specific command-line arguments we used for each algorithm in Supplementary Material.

### 4.2 Evaluation on real data

We perform two sets of experiments on our real datasets: one focusing on varying mismatch tolerance and the other on varying input sizes. We discuss the results in the following.

#### 4.2.1 Evaluation of the impact of mismatch allowance

**Figure 1.**
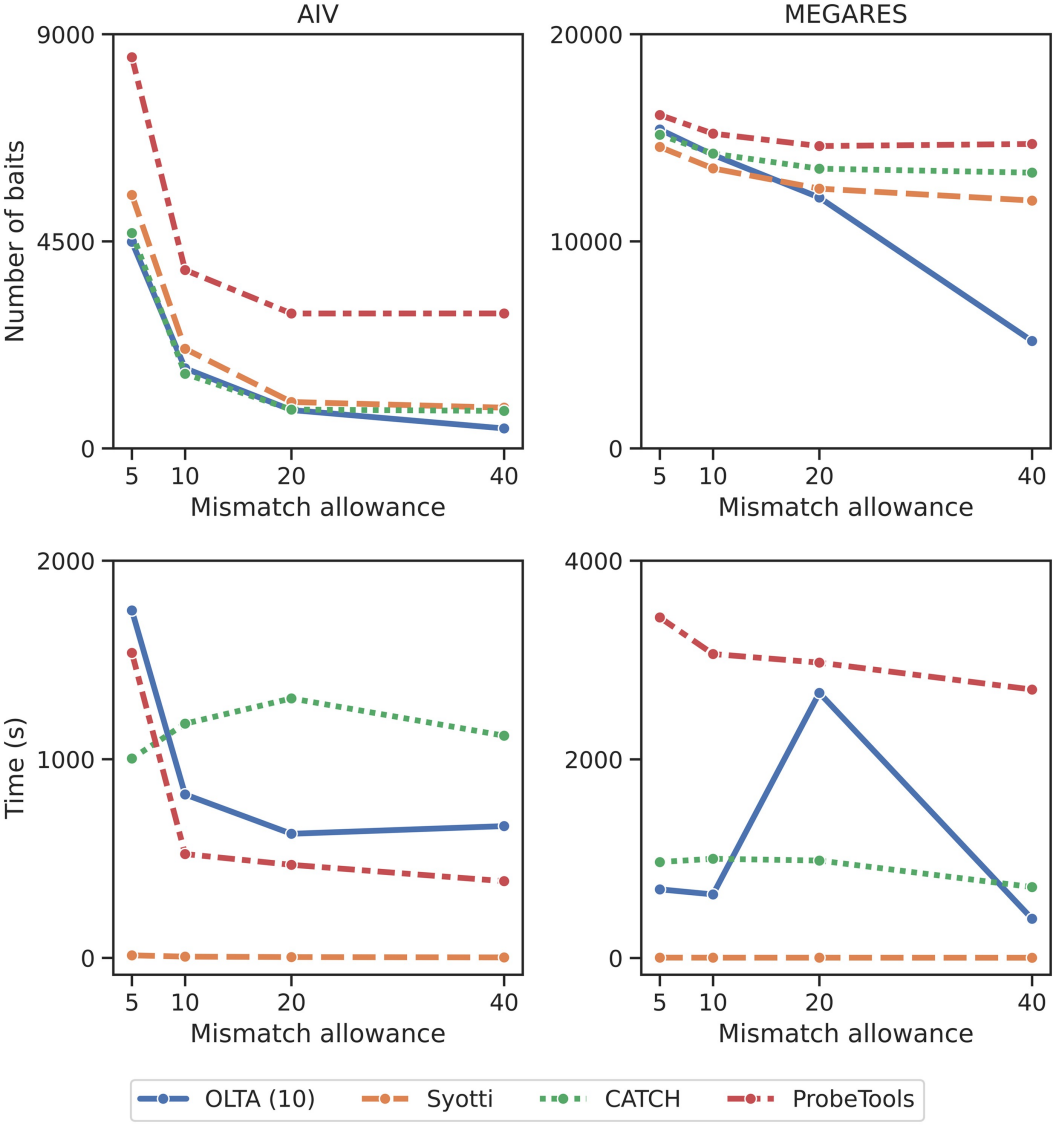
The number of produced baits (top) and running times (bottom) of the four algorithms with varying mismatch tolerances. Experiments were conducted on two input sets containing approximately 8M bases from AIV and 4M bases from MEGARES datasets, respectively. The table form of these results are provided in Supplementary Tables S6 and S7.

One of the factors which influence the success of the bait identification algorithms is the number of mismatches allowed while designing the baits. In the literature, this number varies based on the bait length, with more mismatches allowed with increasing bait length. Our first experiment observes how the maximum mismatch tolerance affects the performance of each method. To measure this, we use two input sets containing approximately 4M bases from MEGARES and 8M bases from AIV datasets, respectively. We set the bait length to 120 bases and ran each method on both inputs with mismatch tolerance of 5, 10, 20, and 40; resulting in 16 experiments per dataset. It is worth noting that 40 mismatches were used in the recent literature as this is recommended by the bait manufacturing company Agilent (Alanko *et al.* 2022). We used OLTA with the parameter ω=10 for these experiments. Figure 1 presents the results.

Our results demonstrate that for mismatch allowance of 20 and 40, OLTA yields the fewest baits among all methods. For smaller mismatch allowances, OLTA, CATCH, and Syotti are sensitive to the input. For AIV dataset OLTA and CATCH yield the least baits, whereas for the MEGARES data, Syotti produce the least baits. As the mismatch allowance increases, OLTA stands out among the four methods for both datasets, particularly for the MEGARES data. This is because OLTA is designed to handle mismatches by representing baits with buckets. In total of eight cases (2 datasets × 4 mismatch allowances), OLTA produces the fewest baits in five cases, Syotti in two cases, and CATCH in only one case, demonstrating that OLTA is the most robust of the four competing algorithms. The running times of all the algorithms tend to go down with increasing mismatch allowance. The performance of OLTA is affected by its ability to create buckets. As a result, we observe that the running time performance of OLTA may rarely go up with more mismatch tolerance, yet all the methods have reasonable running times (<1 h for the most time consuming algorithm, ProbeTools) for this scale of the dataset.

#### 4.2.2 Evaluation of the impact of target sequence length

**Figure 2.**
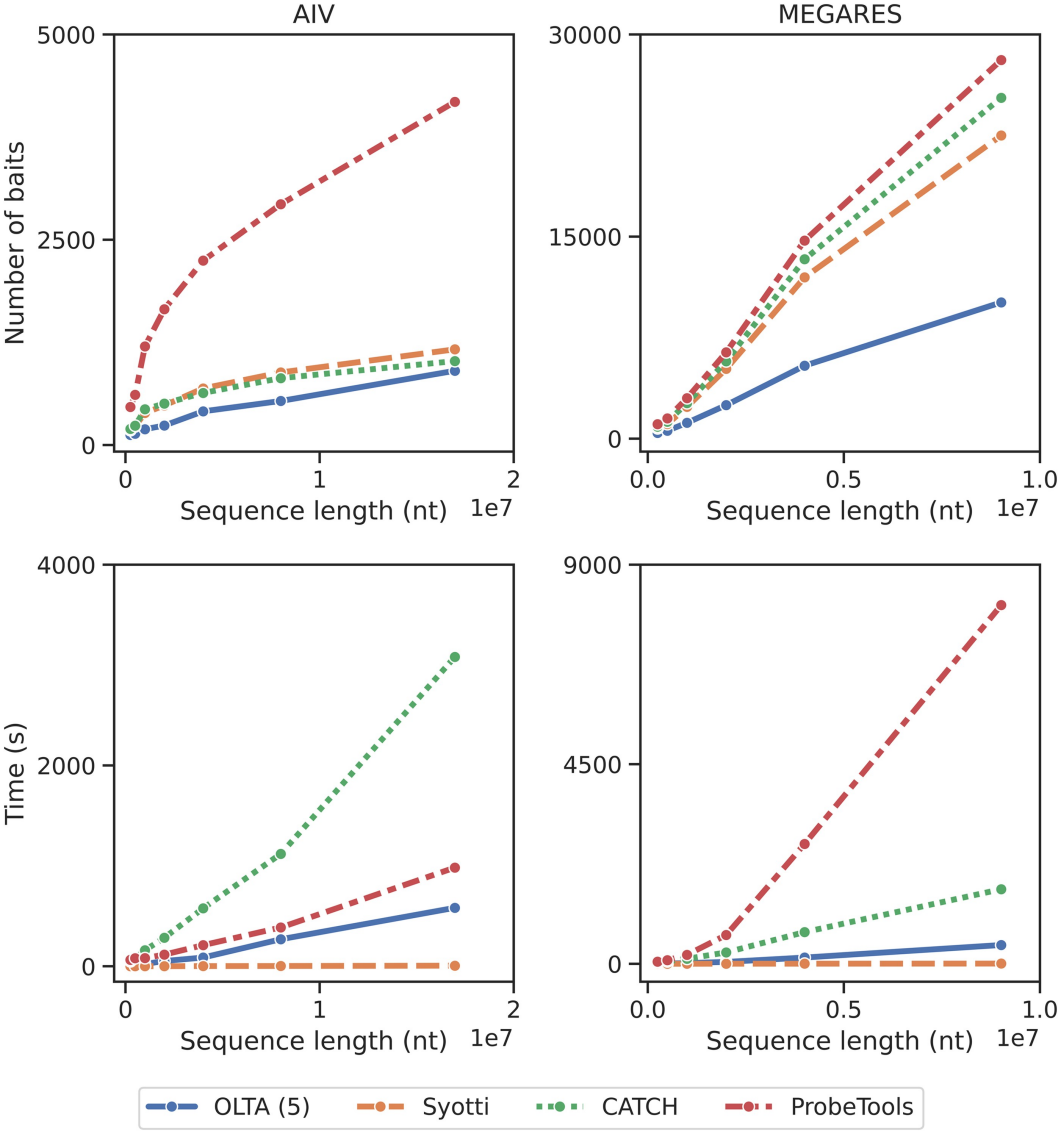
The number of produced baits (top) and running times (bottom) of the four algorithms applied to sets of input sequences from AIV (left) and MEGARES (right) datasets. The table form of these results are provided in Supplementary Tables S1 and S2.

For both real datasets, we use sets of sequences with approximately exponentially increasing total sequence lengths ranging from 250K nucleotides up to the entire dataset. For each total sequence length l, we constructed the input set by sequentially adding sequences from the respective dataset until the total length exceeded l. In the remaining experiments, we configure the algorithms to produce baits with a mismatch tolerance of 40 as this is recommended by the bait manufacturing company, Agilent unless otherwise stated. We ran all methods on all input sets, resulting in 52 experiments [(6 MEGARES sets + 7 AIV sets) × 4 methods] and report the bait numbers and running times in Fig. 2.

Our results demonstrate that when mismatch tolerance is set to 40, for all input sizes, OLTA consistently yields substantially fewer baits than all the competing methods. For AIV sequences, OLTA produced at least 11% and up to 50% fewer baits than the second best algorithm. For MEGARES sequences, OLTA achieved an even better performance, covering the input sequences using 45%–55% fewer baits than the closest competing method. This significant reduction in the number of baits is crucial, as it directly impacts the cost of targeted sequencing. ProbeTools consistently produced the highest number of baits, while Syotti produced fewer baits than CATCH on MEGARES trials, but alternated in performance with CATCH on AIV sequences.

Syotti had the shortest running time in all trials. OLTA was the second-fastest algorithm for three experiments, third fastest in four experiments, and slowest only once for the AIV dataset which happens when the mismatch tolerance is 5. That said, all methods scale to the datasets used in our experiments in <1 h for the slowest case (which is ProbeTools on the MEGARES dataset for 5 mismatch allowance). In summary, OLTA is the most favorable algorithm due to its consistently superior performance in terms of the bait counts while its running time remains acceptable (see Supplementary Tables S1–S9 for all running time results).

#### 4.2.3 Evaluation of the per-bait coverage and redundancy

**Figure 3.**
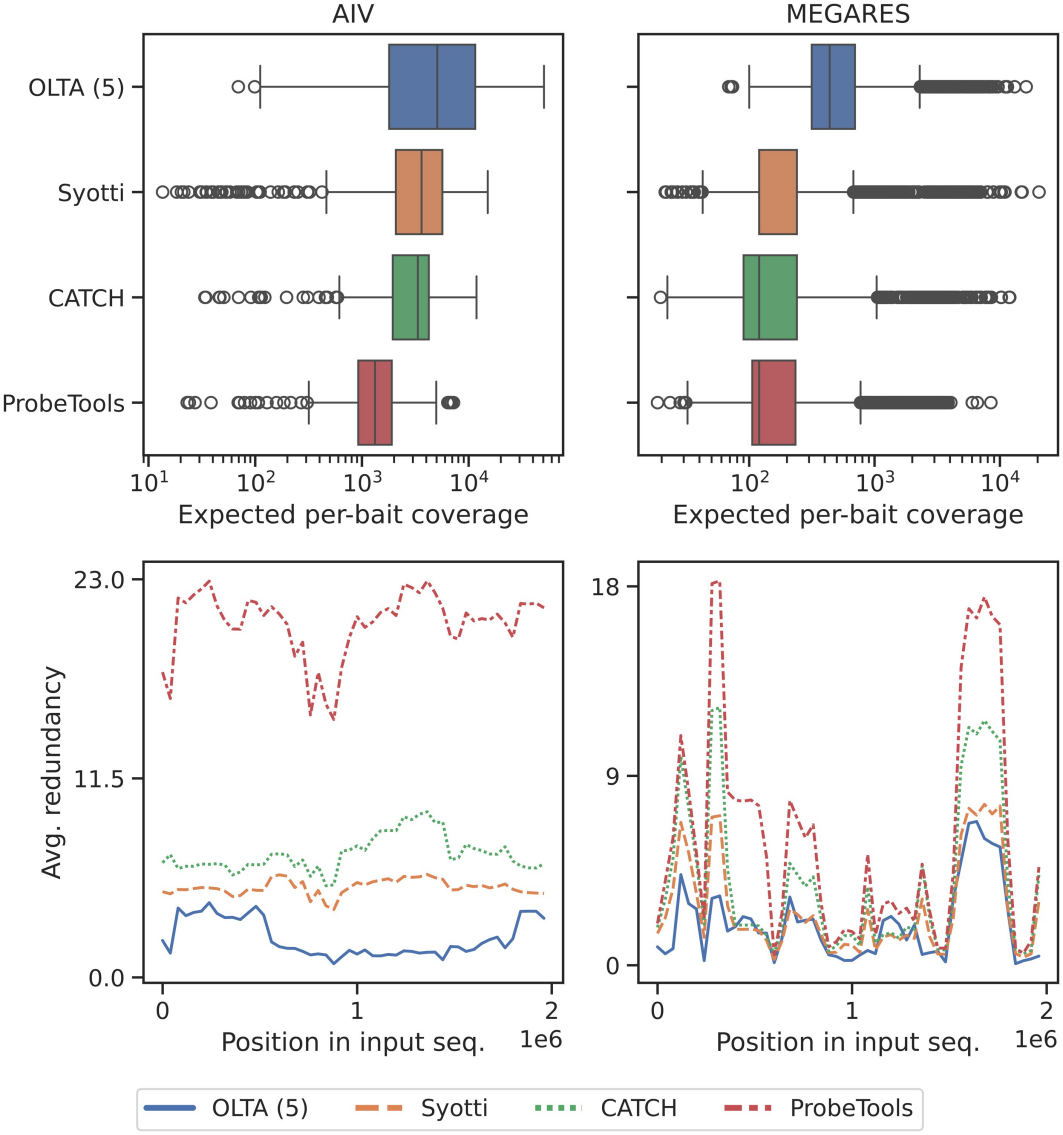
The per-bait coverage (top) and redundancies (bottom) of the four algorithms applied to sets of sequences with 2 million nucleotides. The redundancies were visualized by separating the 2 million positions into 50 roughly equal-length segments and plotting the average redundancy of each segment.

We utilized two sets (one from each dataset) of sequences with approximately two million nucleotides. We report the results in Fig. 3. In both experiments, OLTA produce the bait sets with the highest average per-bait coverage; this is desirable as it demonstrates that OLTA baits are more likely to be utilized in targeted sequencing. It is also expected as the bait per-bait coverage add up to the sequence length and OLTA produces fewer baits than the other algorithms. Additionally, the minimum per-bait coverage of OLTA was two to three times greater than the second greatest one in both experiments. This shows that the baits produced by OLTA are more likely to cover multiple regions with lesser likelihood of bait-bait interference. In terms of redundancy, OLTA’s bait sets demonstrated the lowest mean and maximum redundancies in both experiments, providing additional evidence that the baits produced by OLTA are less prone to interference.

#### 4.2.4 Evaluation of the impact of nucleotide composition

**Figure 4.**
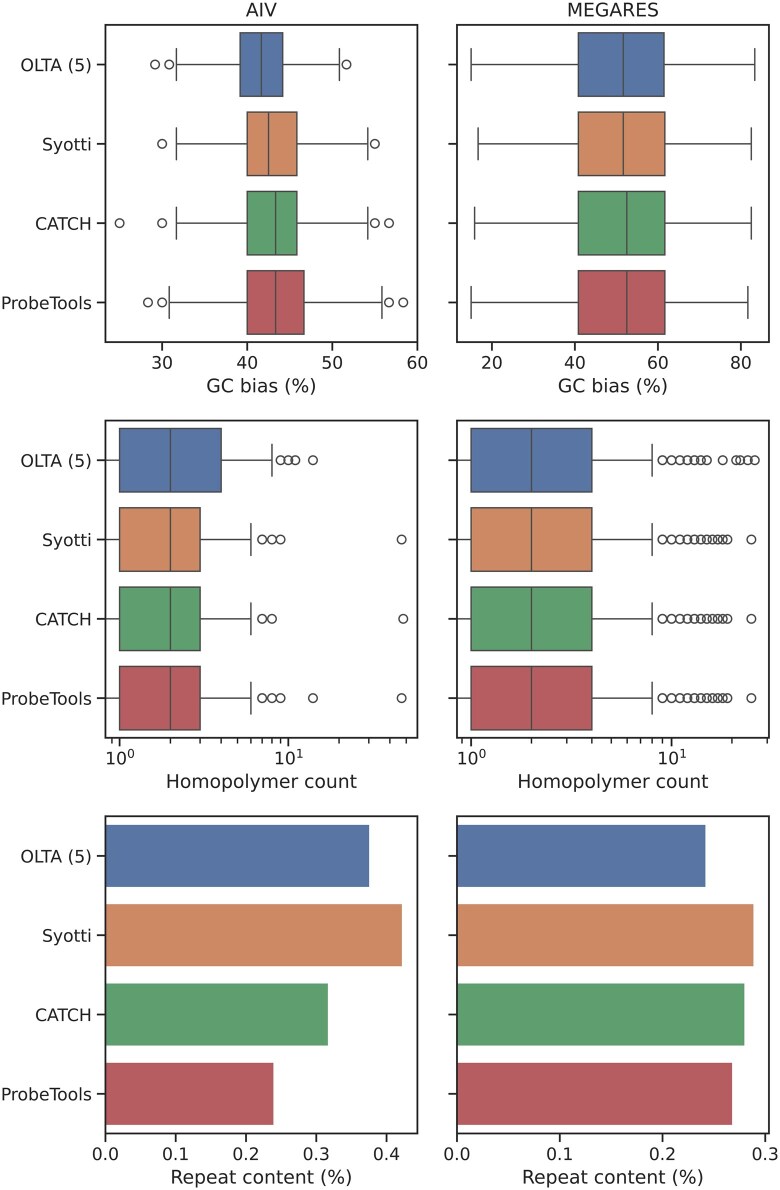
The GC bias (top), homopolymer presence (middle), and repeat content (bottom) of the four algorithms’ outputs on sets of sequences with 2 million nucleotides. A homopolymer was defined as a streak of four identical bases, with longer streaks counting as additional homopolymers (e.g. a streak of five was counted as six homopolymers). Repeat content was calculated using RepeatMasker.

We use the same bait sets to measure the GC bias, homopolymer presence, and repeat content in the algorithms’ outputs. We defined a homopolymer as a streak of 4 identical bases, with longer streaks counting as additional homopolymers (e.g. a streak of 5 was counted as 2 homopolymers). We used RepeatMasker to identify repetitive regions in the produced baits (Smit *et al.* 2013–2015). We report the results in Fig. 4. The algorithms produced similar results in all metrics, suggesting that the baits produced by OLTA would be no less effective than the other algorithms’ outputs in practice.

### 4.3 Evaluation on synthetic data

**Figure 5.**
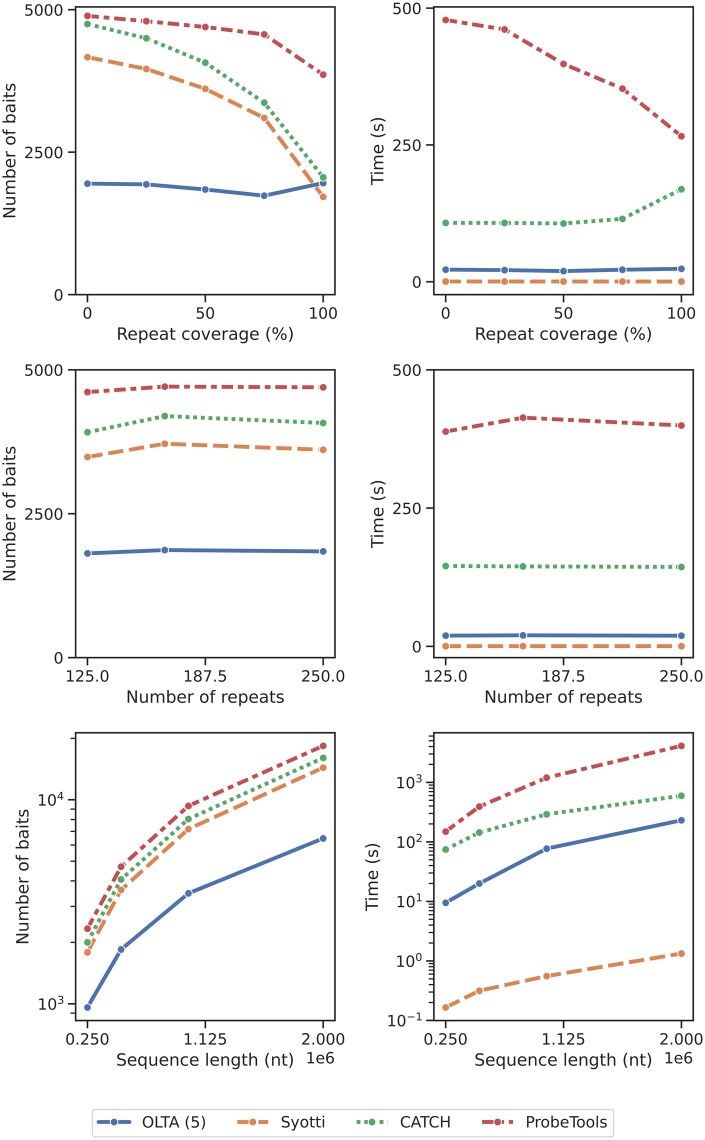
The number of produced baits (left) and the running times (right) of four algorithms applied to synthetic data under various settings. Top: varying repeat coverage (*RC*); middle: varying number of unique repeats (*RN*); bottom: varying input data size (*SL*). The bottom plots are displayed in logarithmic scale. The table form of the results are provided in Supplementary Tables S3 to S5.

To assess the impact of different data characteristics on the performances of the algorithms, we created sequences by independently varying each parameter—repeat coverage (*RC*), number of unique repeats (*RN*), and sequence length (*SL*)—while keeping the other two parameters constant. For the *RC* experiments, we varied *RC* from 0 to 1 in increments of 0.25 while fixing *RN* at 166 and *SL* at 500 000. In the *RN* experiments, we used the *RN* values of 125, 166, and 250, and set *RC* to 0.5 and *SL* to 500 000. For the *SL* experiments, we fixed *RC* at 0.5, varied *SL* with values of 250 000, 500 000, 1 000 000, and 2 000 000, and set *RN* to $\frac{SL}{3}$ for each sequence. We report the algorithms’ resulting bait numbers and running times in Fig. 5 and discuss the results in detail below. In summary, our experiments on the synthetic data suggest that OLTA is a robust method, consistently yielding the smallest bait set for tested data characteristics with the exception of one setting (where the repeat coverage is 100%). Furthermore, OLTA’s running time is consistently the second smallest across all parameter settings.

#### 4.3.1 Impact of repeat coverage

Increasing/decreasing *RC* with a constant number of unique repeats increases/decreases the expected number of copies of each unique repeat in the input data. This variation affects the ease of covering the input sequence with a small number of baits. Here, we examine the performance of each method in response to changes in data characteristics. We observe that OLTA consistently produces the smallest bait set in all experiments except for the extreme case when the entire sequence is covered with repeats (Fig. 5, top row). We hypothesize that this may be an adversarial case which causes the repeats to fall out of alignment with the buckets, causing OLTA to identify them poorly. For low repeat coverage (the harder-to-solve cases), OLTA demonstrates a significant advantage over the other three competing methods. Specifically, OLTA produced fewer than half as many baits as the second-best algorithm on sequences where *RC* was <0.5.

All algorithms produced fewer baits as the sequences included more repetitions (with the exception of OLTA in the last trial). This is expected because as *RC* increases, the likelihood of covering a larger fraction of the input sequence with the same bait increases, making the problem a relatively special case for all algorithms.

Regardless of the repeat coverage, OLTA was consistently ranked as the second fastest algorithm after Syotti, followed by CATCH and ProbeTools. OLTA and Syotti running times were marginally affected by increasing *RC*. In contrast, ProbeTools displayed a substantial decrease in running time, whereas CATCH remained stable until RC=0.75, where it displayed an increase.

#### 4.3.2 Impact of the number of unique repeats

This parameter has a dual effect on data characteristics as compared to the previously discussed *RC*. Increasing/decreasing *RN*, while keeping repeat coverage, decreases/increases the expected number of times that a given repeat pattern iterates in the input data. Consequently, this adjustment makes it harder/easier to cover a large segment of the input sequence with a small number of baits landing on these repeat patterns. We observe the reaction of each method to such changes in data characteristics (Fig. 5, middle row).

In our experiments, this parameter shows a marginal effect on all the four algorithms we test. Consistent with our previous results, OLTA produces fewer baits than all the other methods compared, regardless of the *RN* value. Interestingly, all algorithms produced the most baits when RN=166. We hypothesize that this might be due to the randomization of the nonrepeating regions in the sequences. None of the algorithms’ running times were significantly affected by the number of unique repeats. These results suggest that the scalability of all four algorithms is indifferent to the number of unique repeats in the input sequence.

#### 4.3.3 Impact of sequence length

The purpose of this experiment was to observe how these four algorithms scale with input size. Our experiments on the synthetic data are consistent with those on the MEGARES data. We observe that OLTA consistently outperforms the competing methods across all input sizes. OLTA produces the fewest baits, consistently yielding about half as many baits as the second-best algorithm (Fig. 5, bottom row). As the input size increases, the gap between OLTA and the other methods grows in favor of OLTA. The remaining three algorithms yield comparable numbers of baits, with Syotti producing the fewest, followed by CATCH and then ProbeTools. The number of baits for all algorithms displays an approximately linear trend with increasing sequence length. OLTA is once again the second fastest algorithm after Syotti, followed by CATCH and ProbeTools. It is worth noting that these results reflect the performances of all the methods for the algorithmically hardest case, when the mismatch tolerance is set to the highest value of 40, demonstrating that OLTA’s bucketing approach benefits the most of divergence of the baits as compared to the competing methods.

## 5 Conclusion

Most bait design tools in the literature search for candidate baits within the exact substrings of their input sequences. While this approach allows for quick computation by significantly constraining the candidate options, it also overlooks potential candidates that can cover extensive regions despite not being exact copies of any specific region. To address this limitation, we develop an algorithm, named OLTA.

Our results on real and synthetic datasets demonstrate that, on average, our algorithm yields substantially fewer baits than the compared methods across inputs of varying sizes and characteristics while maintaining practical running time. The improvement is especially notable on harder-to-solve inputs with more randomized content, and also when larger mismatch tolerance is allowed, as our algorithm produces fewer than half as many baits as the second-best algorithm on such sequences. The baits produced by our algorithm also have less redundancy in the regions they cover, greatly reducing the odds of bait interference during hybridization compared to the other algorithms. Our results show that constructing string centers can yield significant improvements over existing methods for designing baits.

In summary, OLTA is able to reduce the number of baits needed for targeted sequencing, which is the main goal of this study, while at the same time increasing utilization (per-bait coverage) of the final bait set and minimizing the bait redundancy. OLTA achieved this while having a practical running time. These results suggest that OLTA has great potential to make targeted sequencing cheaper and more effective, opening up possibilities to study genomes on a larger scale.

OLTA currently has limited customizability as it always uses the Hamming distance criterion for hybridization and aims for complete coverage. Future work would focus on implementing support for different hybridization criteria (e.g. requirement for contiguous matching regions) and target coverage ratios to improve the flexibility of OLTA. OLTA also ensures a covering bait for every nucleotide, but allowing for gaps between baits can further reduce the number of produced baits. In terms of performance, the majority of OLTA’s running time stems from how it adapts WFC-CSP: Closest String focuses on finding a center that minimizes the maximum Hamming distance to any input string, whereas OLTA aims to find a center that is close enough to a maximal subset of the input strings (and whose distance to the strings outside this subset is not of importance). OLTA makes repeated use of WFC-CSP to repurpose it accordingly, resulting in additional computational cost. A modified string center heuristic could improve OLTA’s performance by eliminating this cost.

## Supplementary Material

btaf146_Supplementary_Data

## Data Availability

The data underlying this article are available in Zenodo, at https://doi.org/10.5281/zenodo.15086636. Some datasets were derived from sources in the public domain: (1) AIV, from https://github.com/KevinKuchinski/ProbeTools/commit/275ee2a5d209dc308a3d3e5e8f78ca3861700876#diff-41b6575c39381ee118e740a02d7a9fa3cafd8a3fed4f699e57a5f77e9e9a2186; and (2) MEGARES v3, from https://www.meglab.org/megares/download/.
