# Correction to: OLTA: Optimizing bait seLection for TArgeted sequencing

**DOI:** 10.1093/bioinformatics/btaf505

**Published:** 2025-09-30

This is a correction to: Mete Orhun Minbay, Richard Sun, Vijay Ramachandran, Ahmet Ay, Tamer Kahveci, OLTA: Optimizing bait seLection for TArgeted sequencing, *Bioinformatics*, Volume 41, Issue 4, April 2025, btaf146, https://doi.org/10.1093/bioinformatics/btaf146

In the originally published version of this manuscript, the algorithm description section incorrectly referred to specific lines of the provided pseudocodes. Specifically, there were seven sentences in “3.2 OLTA Algorithm” that used the LaTeX expression *creftype* or *creftypeplural* followed by numbers that do not correspond to the pseudocode line numbers that the sentence refers to. These usages were modified to eliminate the unnecessary LaTeX expressions and refer to the corresponding pseudocodes and line numbers.

