# Supplementary material for: OLTA: Optimizing bait seLection for TArgeted sequencing
Source: Bioinformatics. 2025 Apr 2;41(4):btaf146. doi: 10.1093/bioinformatics/btaf146 (PMC12033030; doi:10.1093/bioinformatics/btaf146)
Supplement: btaf146_Supplementary_Data [file btaf146_supplementary_data.pdf]

Supplementary materials

Claims regarding buckets

Claim: Let $\mathcal{T}^*$ be a set of buckets constructed by Algorithm 2 for some sequences $\mathcal{S}$. A bait set $\mathcal{B}$ that θ-matches every bucket in $\mathcal{T}^*$ will also θ-cover every character in S^*.

Proof We prove the result for a single sequence and its respective buckets. Let S be the input sequence that is to be θ-covered by $\mathcal{B}$. Using the definitions in the algorithm description, the buckets in $\mathcal{T}^*$ are characterized as follows:

$$\mathcal{T}_k^S = \begin{cases} \{S[0 \ldots L]\}, & \text{if } k = 1 \\ \{S[|S| - L \ldots |S|]\}, & \text{if } k = u_S \\ \{S[(k-1)(L-(\omega-1)) + j \ldots (k-1)(L-(\omega-1)) + L + j] : 0 \leq j < \omega\}, & \text{otherwise} \end{cases}$$

Notice that for any bucket $\mathcal{T}_k^S$ that is not the first or last bucket (formally $k \notin \{0, u_S\}$), all substrings in the same bucket overlap $L - (\omega - 1)$ in characters. Specifically, for a bucket $\mathcal{T}_k^S$, all substrings $s^S \in \mathcal{T}_k^S$ span the segment $S[(k-1)(L-(\omega-1)) + (\omega-1) \ldots (k-1)(L-(\omega-1)) + L]$ of S. So a bait that θ-matches $\mathcal{T}_k^S$ will also θ-cover all positions in the segment. We refer to this as the *definite segment of* $\mathcal{T}_k^S$.

Consider two consecutive buckets, $\mathcal{T}_k^S$ and $\mathcal{T}_{k+1}^S$, neither of which are the first or last buckets. Then the ending position the definite segment of $\mathcal{T}_k^S$ can be expressed as:

$$\begin{aligned}(k-1)(L-(\omega-1)) + L &= (k-1)(L-(\omega-1)) + L - (\omega-1) + (\omega-1) \\ &= k(L-(\omega-1)) + (\omega-1)\end{aligned}$$

which is exactly equal to the starting position of the definite segment of $\mathcal{T}_{k+1}^S$. In other words, the definite segments of the buckets $\mathcal{T}_2^S \ldots \mathcal{T}_{u_S-1}^S$ form a contiguous segment $S[L-(\omega-1) \ldots (u_S-2)(L-(\omega-1)) + L]$. Then, θ-matching all of these buckets would θ-cover this segment in S. So all that is left is to θ-cover the segments $S[0 \ldots L-(\omega-1)]$ and $S[(u_S-2)(L-(\omega-1))+L \ldots |S|]$.

Recall that $\mathcal{T}_1^S$ only has a single substring $S[0 \ldots L]$. Then, θ-matching $\mathcal{T}_1^S$ is guaranteed to θ-cover $S[0 \ldots L]$, which includes the segment $S[0 \ldots L-(\omega-1)]$.

The only unused bucket so far is $\mathcal{T}_{u_S}^S$, so we have to show that θ-matching this bucket corresponds to θ-covering our last segment $S[(u_S-1)(L-(\omega-1)) + L \ldots |S|]$. Recall that $u_S = \lceil \frac{|S|}{L-(\omega-1)} \rceil$. Then, the starting position of our last segment can be expressed in two cases:

Case 1: $|S| = u_S \cdot (L-(\omega-1))$, or equivalently $u_S = \left\lceil \frac{|S|}{L-(\omega-1)} \right\rceil = \frac{|S|}{L-(\omega-1)}$

$$\begin{aligned}(u_S-2)(L-(\omega-1)) + L &= \left(\frac{|S|}{L-(\omega-1)} - 2\right) \cdot (L-(\omega-1)) + L \\ &= \left(\frac{|S| - 2(L-(\omega-1))}{L-(\omega-1)}\right) \cdot (L-(\omega-1)) + L \\ &= |S| - 2(L-(\omega-1)) + L \geq |S| - L\end{aligned}$$

Case 2: $|S| = (u_S - 1) \cdot (L-(\omega-1)) + r, r \in \mathbb{Z}^+$ and $0 < r < L - (\omega-1)$

$$\begin{aligned}(u_S-2)(L-(\omega-1)) + L &= \left(\frac{|S| - r}{L-(\omega-1)} - 1\right) \cdot (L-(\omega-1)) + L \\ &= \left(\frac{|S| - r - (L-(\omega-1))}{L-(\omega-1)}\right) \cdot (L-(\omega-1)) + L \\ &= |S| - r - (L-(\omega-1)) + L \geq |S| - L\end{aligned}$$

In either case, the starting point of this segment occurs later than $|S| - L$, which is the starting point of the only substring in $\mathcal{T}_{u_S}^S$. So θ-matching $\mathcal{T}_{u_S}^S$ would θ-cover this segment and in turn the entire sequence. □

Claims regarding Hamming distance

Claim: For a positive integer θ and two strings T and T' such that $d(T, T') \leq 2\theta$, there exists a string B such that $d(T, B) \leq \theta$ and $d(T', B) \leq \theta$.

Proof If $d(T, T') \leq \theta$, then T is a string that satisfies this property as $d(T, T) = 0$ and $d(T, T') \leq \theta$. Assuming $\theta < d(T, T') \leq 2\theta$, consider the positions in which T and T' have different characters, namely $M = \{j : T[j] \neq T'[j]\}$. We write M as a sequence $j_1, j_2, \ldots, j_{|M|}$ and assign the characters of B as follows:

$$B[i] = \begin{cases} T[i], & \text{if } \exists k \text{ such that } i = j_k \text{ and } k \leq \theta \\ T'[i], & \text{otherwise} \end{cases}$$

Since $T[i] = T'[i]$ for any $i \notin M$, the mismatching positions of B and T' are the positions in M that have been set to match with T. Since there are exactly θ such positions, $d(B, T') \leq \theta$. Similarly, the mismatching positions of B and T are the positions in M that have been set to match with T'. There are $|M| - \theta$ such positions, where $|M| \leq 2\theta$. Then $d(B, T) \leq \theta$ as well. □

Claim: For a positive integer θ and two string T and T', if there is a string B that satisfies $d(B, T) \leq \theta$ and $d(B, T') \leq \theta$ then $d(T, T') \leq 2\theta$.

Proof Consider the sets $M_T = \{i : B[i] \neq T[i]\}$ and $M_{T'} = \{j : B[j] \neq t[j]\}$ which contain the mismatching positions of B and T and B and T' respectively. Notice if a position k is not in either of these, $T[k] = B[k] = T'[k]$. By contrapositive, if $T[k] \neq T'[k]$ for some k, then $k \in M_T \cup M_{T'}$. Recall that $d(T, T') = |\{k : T[k] \neq T'[k]\}| = |M_T \cup M_{T'}| \leq |M_T| + |M_{T'}|$, where $|M_T| \leq \theta$ and $|M_{T'}| \leq \theta$. So $d(T, T') \leq 2\theta$. □

Analyzer implementation and details

Unlike other bait analyzers for targeted enrichment, our analyzer does not consider reverse complements and computes bait alignments using the naïve algorithm for the SLIDING WINDOW HAMMING DISTANCES PROBLEM [Navarro, 2001] rather than the conventional seed-and-extend heuristic [Baeza-Yates and Perleberg, 1996]. We observed that seed-and-extend alignment vastly underestimates the coverage of our algorithms' solution sets. This discrepancy is likely due to our use of WFC-CSP, which results in baits that lack contiguous exact matches suitable for use as seeds.

We also experimented with the standard FFT-based algorithm [Fischer and Paterson, 1974] due to its reported performance on small alphabets [Baba et al., 2003], but found that the naïve algorithm runs faster for the solution sets produced by our experimental setup.

Synthetic sequence generation algorithm

We generate the synthetic sequences as follows: For a given sequence length SL, we start by initializing a vector of that length filled with placeholder characters. We also randomly generated RN strings with lengths between 120 and 240 (L to $2L$) over the alphabet $\Sigma =$ {A, G, T, C}. We refer to these strings as "unique repeats" and use them to populate the vector. Until a desired fraction RC of our vector is populated, we pick the next random unique repeat and produced an imperfect copy of it by modifying up 40 of its characters. We plant the resulting copy in a random unpopulated section of the vector and repeated this process. Once we populate the desired fraction RC with imperfect repetitions, we randomize the remaining placeholder characters over Σ to finalize the sequence.

Algorithm 5 Sequence synthesizing

Input: Sequence length SL, repeat number RN, repeat coverage RC
Output: Sequence S

$S \leftarrow \underbrace{\epsilon\epsilon\ldots\epsilon}_{SL \text{ times}}$ ▷ Initialize S with SL placeholder character ϵ
$s^* \leftarrow [s_1, s_2, \ldots s_{RN}]$ where $\forall i : 1 \leq i \leq RN, s_i = \epsilon$ ▷ List of short seed sequences
for $i = 1$ **to** RN **do**
  $l \leftarrow$ Random(120, 240) ▷ Randomize seed sequence length
  $s_j \leftarrow$ a random sequence over the alphabet $\Sigma = \{A, G, T, C\}$ with length l
end for
$i \leftarrow 1$
while $\frac{|\{j:S[j]\neq\epsilon\}|}{SL} \leq RC$ **do** ▷ Until RC of S is populated with seed sequences
  $p \leftarrow$ Random$(0, SL - 1)$ ▷ Pick a position in S to plant s_i
  while $|\{j : p \leq j < |s_i| \text{ and } S[j] = \epsilon\}| > 0$ **do**
    $p \leftarrow$ Random$(0, SL - 1)$ ▷ If planting s_i here would overwrite a non-ϵ character, pick another position
  end while
  $\theta \leftarrow$ Random$(0, 40)$ ▷ Modifications to make on s_i before planting it in S
  $M \leftarrow \theta$ distinct random integers in the range $[0, |s_i| - 1]$
  for $j = 0$ **to** $|s_i| - 1$ **do**
    if $j \in M$ **then**
      $S[p + j] \leftarrow$ random letter from $\Sigma \setminus s_i[j]$
    else
      $S[p + j] \leftarrow s_i[j]$
    end if
  end for
  $i \leftarrow i \pmod{RN} + 1$
end while
return S

Algorithm configurations

The following are the optional arguments we provided when running each algorithm. The details for each argument can be found in the respective manuscript/repository of each algorithm. As a reminder, each algorithm was allowed to parallelize over 8 processes.

- Syotti: `--randomize --n-threads 8`
- CATCH: `--probe-stride 60 --max-num-processes 8 --filter-with-lsh-minhash 0.6 --cluster-and-design-separately 0.15 --cluster-from-fragments 50000`. We used the last three arguments as suggested by the authors.
- ProbeTools: `-c 100 -k 120 -l 120 -L 1 -T 8`. It should be noted that ProbeTools uses BLAST to compute bait alignments. As a result, its hybridization criteria is slightly different than the other three algorithms as it (i) expects a sequence identity threshold instead of a Hamming distance threshold, (ii) looks for local alignments/allows "overhanging" bases for alignments, and (iii) allows for insertions/deletions. We provided the identity threshold by dividing the number of required matches by the probe length, *e.g.* a mismatch allowance of 40 with a bait length of 120 would result in a 0.66 sequence identity. We also used the `-l` flag to minimize "overhanging" alignments by enforcing bait-target alignments to consist of at least 120 base pairs.
- OLTA: `-t 8`.

Redundancy and per-bait coverage definitions

Redundancy: Given a bait set $\mathcal{B}$, a position i in a sequence S, and a mismatch allowance θ we define the *covering baits* of i in S as the set of baits in $\mathcal{B}$ that θ-cover i in S. If a position i in S has $k > 0$ covering baits, we say that $\mathcal{B}$ has $k - 1$ *redundancy* at that position, formally defined as:

$$R_\theta(\mathcal{B}, S, i) = \max(0, |\{B \in \mathcal{B} : B\ \theta\text{-covers } i \text{ in } S\}| - 1)$$

In particular, a redundancy of 0 on a position means that the bait set has at most one bait covering that position. Low redundancy is desirable, as it reduces the likelihood of interference among baits and ensures that trimming the solution set would not harm coverage.

Per-bait coverage: Given a bait B in a bait set $\mathcal{B}$, a position i in a sequence S, and a mismatch allowance θ, we define a *hybridization polynomial* as a first-degree polynomial $P_\theta(B, \mathcal{B}, S, i) = x \cdot \alpha + (1 - \alpha)$, where α is the probability that B hybridizes with S to cover position i given θ and $\mathcal{B}$. We take the product of a bait's hybridization polynomials across every position and every sequence from a set of input sequences $\mathcal{S}$ to calculate the *cumulative hybridization polynomial* $P^*_\theta(B, \mathcal{B}, \mathcal{S}) = \prod_{S\in\mathcal{S}} \prod_{i=0}^{|S|-1} P_\theta(B, \mathcal{B}, S, i) = \beta_0 + \beta_1 x + \beta_2 x^2 + \cdots + \beta_n x^n$, where $n = \sum_{S\in\mathcal{S}} |S|$ and β_i represents the probability of B covering an exact total of i positions in $\mathcal{S}$. Based on this polynomial, we define the *expected per-bait coverage* of B in $\mathcal{B}$ on

$\mathcal{S}$ as the expected number of positions to be covered by B in $\mathcal{S}$:

$$\mathbb{E}_\theta(B, \mathcal{B}, \mathcal{S}) = \sum_{i=0}^{n} i \cdot \beta_i$$

To simplify the calculation of this expression, we assume:

1. Each position that is θ-covered will hybridize with exactly one bait in the solution set. (In reality, this is difficult due to interference between baits *i.e.* if two baits hybridize with less than L nucleotides between them, there is no room for another bait to hybridrize with the remaining nucleotides.)
2. Hybridizations are independent events, *i.e.* a bait hybridizing to a position does not influence which baits will hybridize to other positions.
3. For a position in a sequence, every bait that θ-covers a position is equally likely to hybridize to that position (again, this is not true because the similarity between the region and the bait influences the likelihood of hybridization). Baits that do not θ-cover a position have zero probability of hybridizing to that position.

Given these assumptions, we can characterize the hybridization probability α in any hybridization polynomial $P_\theta(B, \mathcal{B}, S, i) = \alpha \cdot x + (1 - \alpha)$ as just:

$$\alpha := \begin{cases} \frac{1}{|\{B' \in \mathcal{B} : B' \theta\text{-covers } i \text{ in } S\}|}, & \text{if } B \text{ } \theta\text{-covers } S[i] \\ 0, & \text{otherwise} \end{cases}$$

The calculation of the cumulative hybridization polynomial and expected coverage follow from this characterization.

Further time-complexity optimizations

These novel ideas are not implemented in the algorithm due to their late discovery in the research process. We leave these in the paper for future researchers to build off of.

Hamming Distance in constant time with bit hack: Assuming we have an alphabet of size 4 (ATCG), we can use the following strategy:

1. Given a segment of length L, called X, create two bit arrays with length L. We will call these arrays X_1 and X_2.
2. For each index in the nucleotide sequence, if it is A or T, the corresponding index in X_1 gets a 0, otherwise it gets a 1. For each index in the nucleotide sequence, if it is A or C, the corresponding index in X_2 gets a 0, otherwise it gets a 1. For example, ATCG generates X_1=0011 and X_2=0101, and GTAG generates X_1=1001 and X_2=1101. These bit arrays can be pre-computed and stored.
3. The hamming distance between two sequences X and Y is the number of 1 bits in $((X_1 \oplus Y_1)\&(X_2 \oplus Y_2))$, where $\oplus$ is the XOR operator. For example, the hamming distance of ATCG and GTAG is number of 1s in $((0011 \oplus 0101)\&(1001 \oplus 1101)) = (0110\&0100) = 0110$, which has two 1s, hence a hamming distance of 2. Note that the result has 1s where the nucleotides are different and 0s where they are the same.

The number of 1 bits can be handled in C++ with __builtin_popcount() from the GCC compiler. C++ 21 vector<bool> does not support efficient boolean operators, but this can be done with bitset, with the caveat that L needs to be known at compile time. Stitching together long longs or using the boost library's dynamic bitset can be used to get around this caveat. To account for N, we can bitwise or our sequence with a bitmask containing 1s for the locations of N and 0 otherwise.

Faster WFC-CSP: The implementation in [Xu and Perkins, 2022] with S strings each of length L has time complexity $O(L^2)$ assuming S<L. We can reduce it down to $O(S * LlogL)$. For each sequence X in S, we store a sorted vector of pairs: {amount of sequences in S that share the same nucleotide with X at the index, index}. This part takes O($S * LlogL$). We then have sliding pointers for each vector. When a nucleotide in index Z gets placed in the final string, we start sliding each pointer forward if the index (the second value in the pair) is equal to Z. We stop sliding when the pointer points to a value where the index (again the second value in the pair) has not been decided in the final string. In total, we slide S pointers L times each, for a time complexity of $O(S * L)$, which is less than the sorting step.

Experiment results in tabular form

Input size	OLTA (5)		ProbeTools		Syotti		CATCH	
	Time (s)	Baits	Time (s)	Baits	Time (s)	Baits	Time (s)	Baits
251,188	2.17	123	64.331	464	0.094	182	50.424	196
500,437	3.868	137	80.438	611	0.143	228	80.542	235
1,000,981	22.37	192	80.938	1200	0.254	390	159.124	435
2,000,333	51.805	237	115.46	1653	0.494	481	283.11	504
4,000,609	86.679	410	209.771	2246	1.58	689	576.837	634
8,001,286	268.645	536	386.168	2932	2.402	884	1118.552	814
16,971,291	581.81	904	982.006	4179	5.618	1165	3079.862	1022

Table SM. 1. Experiment results on the AIV dataset with increasing input sizes. Mismatch allowance is set to 40.

Input size	OLTA (5)		ProbeTools		Syotti		CATCH	
	Time (s)	Baits	Time (s)	Baits	Time (s)	Baits	Time (s)	Baits
250,051	2.867	421	47.145	1066	0.218	769	35.234	882
500,160	6	583	82.109	1500	0.282	1077	55.676	1248
1,000,807	15.194	1169	200.794	3000	0.466	2357	113.142	2633
2,000,462	42.009	2482	647.897	6400	1.335	5177	255.225	5735
4,000,098	140.489	5411	2702.159	14700	1.927	11967	713.512	13314
9,014,529	422.8	10111	8086.273	28100	4.245	22500	1680.579	25299

Table SM. 2. Experiment results on the MEGARES dataset with increasing input sizes. Mismatch allowance is set to 40.

Input size	OLTA (5)		ProbeTools		Syotti		CATCH	
	Time (s)	Baits	Time (s)	Baits	Time (s)	Baits	Time (s)	Baits
250,000	9.458	959	148.744	2333	0.165	1787	74.389	2000
500,000	19.935	1845	390.749	4696	0.315	3611	143.388	4067
1,000,000	76.931	3477	1196.416	9325	0.555	7186	290.446	8047
2,000,000	230.082	6464	4102.262	18374	1.33	14364	593.993	16032

Table SM. 3. Experiment results on synthetic sequences with increasing sequence lengths. Mismatch allowance is set to 40.

Coverage	OLTA (5)		ProbeTools		Syotti		CATCH	
	Time (s)	Baits	Time (s)	Baits	Time (s)	Baits	Time (s)	Baits
0.0	21.948	1948	478.336	4891	0.306	4167	107.407	4749
0.25	21.149	1935	460.874	4798	0.292	3960	107.342	4498
0.5	19.291	1845	397.936	4696	0.289	3611	106.326	4070
0.75	21.794	1736	352.834	4566	0.282	3101	114.7	3368
1.0	23.577	1958	265.814	3860	0.204	1716	168.895	2058

Table SM. 4. Experiment results on synthetic sequences with increasing repeat coverages. Mismatch allowance is set to 40.

# of seeds	OLTA (5)		ProbeTools		Syotti		CATCH	
	Time (s)	Baits	Time (s)	Baits	Time (s)	Baits	Time (s)	Baits
125	19.312	1810	388.329	4613	0.296	3485	145.147	3915
166	19.939	1868	413.443	4708	0.302	3715	144.48	4196
250	19.154	1845	399.361	4696	0.292	3611	143.524	4076

Table SM. 5. Experiment resuls on synthetic sequences with increasing number of seed repetitions. Mismatch allowance is set to 40.

Mismatches	OLTA (10)		ProbeTools		Syotti		CATCH	
	Time (s)	Baits	Time (s)	Baits	Time (s)	Baits	Time (s)	Baits
5	1749.051	4490	1535.473	8500	12.876	5507	1003.699	4678
10	822.863	1739	522.785	3876	6.28	2163	1178.95	1621
20	624.746	833	468.375	2931	4.15	1008	1306.241	843
40	663.339	433	386.168	2932	2.402	884	1118.552	814
**Average**	965.000	**1873.75**	728.200	4559.75	**6.427**	2390.50	1151.860	1989.00

Table SM. 6. Experiment results on the AIV dataset with increasing mismatch tolerances. We use the second largest input size (i.e., 8M bases) for this dataset.

Mismatches	OLTA (10)		ProbeTools		Syotti		CATCH	
	Time (s)	Baits	Time (s)	Baits	Time (s)	Baits	Time (s)	Baits
5	690.397	15400	3427.582	16100	2.979	14555	965.369	15145
10	639.481	14175	3059.681	15200	2.562	13519	998.248	14236
20	2668.468	12113	2972.819	14600	2.473	12543	980.388	13505
40	394.856	5187	2702.159	14700	1.927	11967	713.512	13314
**Average**	1098.301	**11718.75**	3040.560	15150.00	**2.485**	13146.00	914.379	14050.00

Table SM. 7. Experiment results on the MEGARES dataset with increasing mismatch tolerances. We use the second largest input size (i.e., 4M bases) for this dataset.

Running time with smaller mismatch tolerance

Input size	OLTA (10)	ProbeTools	Syotti	CATCH
251188	19.6	25.322	0.232	51.683
500437	42.299	45.638	0.489	90.016
1000981	98.687	85.5	0.588	173.566
2000333	187.252	180.568	1.173	312.462
4000609	444.13	348.475	2.945	625.771
8001286	1020.77	715.005	5.854	1042.095
16000573	2297.207	1571.472	17.492	2311.13
16971291	2538.92	1634.634	17.025	2595.915

Table SM. 8. Running times of the different algorithms applied to the AIV dataset with 10 mismatches.

Input size	OLTA (10)	ProbeTools	Syotti	CATCH
251188	19.6	25.322	0.232	51.683
500437	42.299	45.638	0.489	90.016
1000981	98.687	85.5	0.588	173.566
2000333	187.252	180.568	1.173	312.462
4000609	444.13	348.475	2.945	625.771
8001286	1020.77	715.005	5.854	1042.095
16971291	2538.92	1634.634	17.025	2595.915

Table SM. 9. Running times of the different algorithms applied to the MEGARES dataset with 10 mismatches.

Results of different OLTA configurations

We picked OLTA-05 as our comparison against other algorithms because it generated fewer baits and ran relatively fast compared to the other algorithms. However, our algorithm is much more robust than that. There is a bait-time trade-off that can be adjusted depending on whether there is a need to reduce baits or reduce the running time by changing the bucket size. As shown in Figure SM. 1 and Figure SM. 2, for our testing configuration of bait length $L = 120$ and mismatch tolerance $\theta = 40$, the minimum number of baits occurs at around OLTA-30. Run time is the least at OLTA-1 and is positively correlated with bucket size. Note that OLTA-40 often produced more baits with a longer runtime than OLTA-30, so we did not include it in our graphs. For all other input variables held constant, bucket size vs number of baits has a very messy U shape. The reason for this is if the bucket size is too small, many of the subsequences of the original input are not considered. If the bucket size is too large, then baits can highly overlap and be redundant. The location of the trough is dependent on factors like the size and repetitiveness of the data set (in Fig 5, we can see that OLTA-20 outperforms 30 at a repeat coverage of 1.0), as well as the bait length and mismatch tolerance. Some informal testing with bait length $L = 60$ and mismatch tolerance $\theta = 6$ on AIV show that the number of baits is the least around OLTA-10. There is no easy/nice mathematical formula to determine where the trough of the U is located; this is analogous to a black box machine learning algorithm where it is hard to predict how hyper-parameters will perform without actually running it.

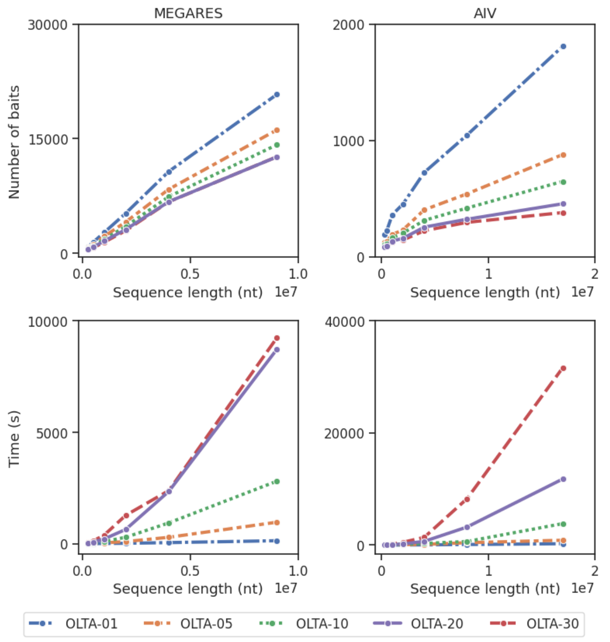

Fig. SM. 1. The number of produced baits (top) and the running times (bottom) of different OLTA configurations applied to MEGARES (left) and AIV (right) sequences. For each plot entry, the number after "OLTA-" represents the ω value of the configuration.

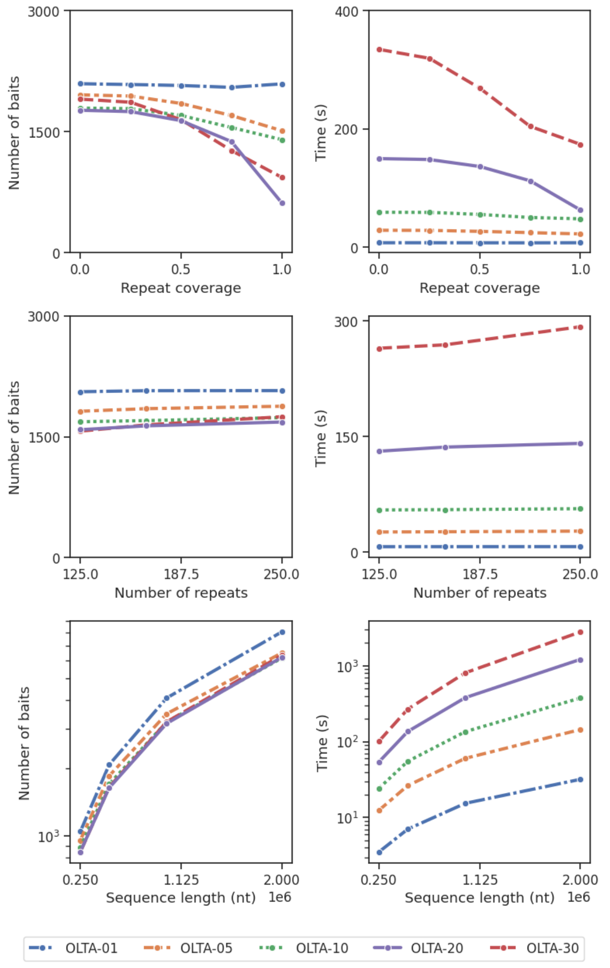

Fig. SM. 2. The number of produced baits (left) and the running times (right) of different OLTA configurations applied to synthetic data under various settings. Top: varying repeat coverage (RC); middle: varying number of unique repeats (RN); bottom: varying input data size (SL). For each plot entry, the number after "OLTA-" represents the ω value of the configuration.
